# An Information Retrieval Approach for Robust Prediction of Road Surface States

**DOI:** 10.3390/s17020262

**Published:** 2017-01-28

**Authors:** Jae-Hyung Park, Kwanho Kim

**Affiliations:** 1 ICT Convergence R & D Center, Metabuild Co., Ltd., 5F 1487-6 Seocho-3dong, Seocho-gu, Seoul 06708, Korea; jhpark@metabuild.co.kr; 2 Department of Industrial and Management Engineering, College of Engineering, Incheon National University, Incheon 22012, Korea

**Keywords:** road surface state detection, road surface radar, smart highway, information retrieval, machine learning, ranking and scoring functions, exponential moving average

## Abstract

Recently, due to the increasing importance of reducing severe vehicle accidents on roads (especially on highways), the automatic identification of road surface conditions, and the provisioning of such information to drivers in advance, have recently been gaining significant momentum as a proactive solution to decrease the number of vehicle accidents. In this paper, we firstly propose an information retrieval approach that aims to identify road surface states by combining conventional machine-learning techniques and moving average methods. Specifically, when signal information is received from a radar system, our approach attempts to estimate the current state of the road surface based on the similar instances observed previously based on utilizing a given similarity function. Next, the estimated state is then calibrated by using the recently estimated states to yield both effective and robust prediction results. To validate the performances of the proposed approach, we established a real-world experimental setting on a section of actual highway in South Korea and conducted a comparison with the conventional approaches in terms of accuracy. The experimental results show that the proposed approach successfully outperforms the previously developed methods.

## 1. Introduction

**Figure 1:**
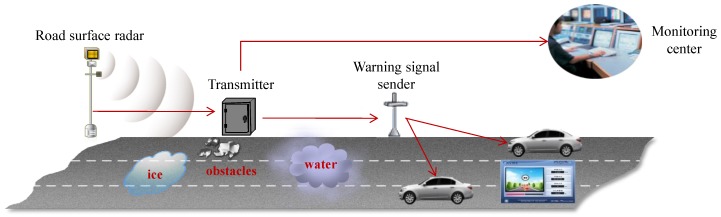
Concept of a smart highway involving the automatic identification of road surface states and the transmission of such information to related systems, such as monitoring centers and nearby vehicles.

There have recently been intensive efforts on automatically identifying road surface conditions using radar sensor technologies without human intervention [1,2,3]. Contrary to the conventional methods that depend heavily on visual information involving human experts, radar-signal based identification of road surface conditions enables not only significantly improved performances, but also automated pre- and post-actions based on the identification results [4]. For instance, the identified road surface conditions in a specific area are delivered to a road monitoring system, and such information is able to be utilized to provide alerts to vehicles near that area, especially when the road conditions are poor for driving, such as for wet and snowy conditions. Figure 1 shows a representative application of a radar-based identification system.

The identification of road surface conditions based on radar signals has become quite valuable, particularly for highway areas, because unexpected changes in road surface conditions often result in severe accidents [5]. In particular, the unexpected road surface conditions on curves, or before and after tunnel areas, are extremely dangerous. Although previously developed methods for road surface identification have been widely adopted in many urban areas, they mainly depend on visual information obtained from closed-circuit television [6]; conventional visual-based methods are ineffective in capturing the road-surface conditions in many cases in terms of weather conditions and time. Those are usually unavailable at night or under heavy rainfall conditions, which cause more severe problems on driver safety.

However, by utilizing radar signals, automatically identifying road surface states is considered a challenging problem owing to the time dependency among the signal data. First, the identified states of a road surface within a particular area are dependent on each other at particular times, which means that each consecutive road surface condition directly affects the next series of conditions. In addition, road surface conditions do not dramatically change within a short period but change quite slowly in most cases. In detail, the reason why we introduce the concept of robustness is to measure how much a detection model is capable of yielding stable results over time. There exists a trade-off between the two concepts, preciseness and robustness, since more robustness means fewer changes of decision. For instance, when the state of the road surface rapidly changes, a model with high robustness is not capable of accommodating such changes.

Next, in a real-world setting, the signals obtained from an installed radar system are highly likely to produce a large amount of noise compared to a similar system installed in a laboratory setting. Such noise is often caused by various un-controllable aspects, such as vehicle movements, objects on the road, and dust in the air, which eventually generate signal interferences. Furthermore, the weather conditions do not necessarily determine the road surface conditions. This means that the accurate road surface conditions in an area cannot be affordable based only on the weather condition in that area. For instance, road surfaces often remain to be in a wet state although the weather condition in the area is sunny, which might be caused by the other factors, such as shadows over the area.

Moreover, two requirements of road surface identification models are needed to be satisfied before such models can be successfully applied in an actual setting. First, such models not only need to be fast, but also sufficiently accurate. Although many effective analysis methods considering time have been developed, they are limited in their application in a real-world setting owing to their high computational complexity. Second, such models need to yield explainable results for adaptive parameter settings. Whenever a model provides the state of a particular road surface, the information regarding its determination should be easily interpretable by human experts as needed for the purpose of fine-tuning and auditing the system in the case of estimation failures.

Therefore, in this paper, we propose an information-retrieval approach towards the robust identification of road surface conditions by combining the previously studied data-mining methods. Specifically, unlike the previously developed approaches that solely utilize signal processing for extracting valuable features and machine-learning methods for finding repeatedly occurring patterns, our approach focuses on yielding precise and robust results by incorporating information retrieval, sophisticated similarity functions, and the moving average method.

When the proposed approach, based on radar signals, is compared with the conventionalimage-based methods, shown in [2,4,6], it is capable of identifying road surface states regardless of weather conditions. The well-known limitation of image-based methods is the fact that the captured images of the road surface cannot be recognizable, especially in dark and rainy conditions. Moreover, obtaining high-resolution images that are necessary to improve the identification performance is quite costly due to the needs of high bandwidth network facilities.

On the other hand, the proposed approach also has some benefits compared to the previously developed signal-based identification methods. With the proposed approach, is not only easy to understand the final results, but also simple to calibrate based on the environments of actual road circumstances. These advantages are crucial for an identification method of road surface states in order to be installed in various conditions in terms of road types, such as highways under specific climates. For instance, when an identification method is set up in a specific area, it is natural that the observation patterns are very differ from those in other settings.

The experimental results based on a real-world setting show that the proposed methods yield quite satisfactory results for detecting road surface conditions in terms of four states, such as dry, snowy, unknown, and wet. Although there is no dominant method across the various experimental settings, on average, the support vector-based method resulted in the best performance, especially for dry and wet states. Additionally, the moving average-based profiling was helpful for better results in the most cases, and it was also beneficial for producing stable results over time.

The remainder of this paper consists of the following five sections: First, in Section 2, previous studies related with the automatic detection of road surface conditions are summarized, and their potential limitations in terms of the problem addressed herein are suggested. Moreover, we describe the underlying rationale and contributions of our research through a comparison with previous studies. Second, in Section 3, we formally define our problem and explain the core concepts of the proposed approach. Next, the dataset used in our research and the performance of the proposed approach are demonstrated in Section 4. Finally, we provide some concluding remarks regarding our research in Section 5.

## 2. Related Works

Many previous studies have aimed to discover the potential benefits of detecting road surface conditions by employing sensing technologies, such as radio frequencies, environmental information, and radar signals. However, such previous studies have mainly focused on how such sensing technologies are able to be adapted to automatically determine the state of a road surface without involving manual tasks by human experts.

Classification models based on machine-learning technologies that utilize radar signals have been reported. In [7], the authors proposed the use of mutual information and fuzzy support vector machines for recognizing radar signals. They focused on extracting effective feature vectors from signals for multi-class pattern recognition problems. A radial kernel function was proposed in [8]. In this study, one-against-one or one-against-rest of a multi-class classifier and parameter selection method were suggested.

In particular, several studies addressing the state identification problem of a road surface have been reported. Most studies in this area have mainly focused on the benefits of specific frequency bands used for the radar system and their effectiveness at various distances. The backscattering properties of asphalt under different conditions have been discovered, and the capabilities of a 24 GHz forward-looking monostatic radar system have been suggested [1]. Moreover, sophisticated signal processing techniques were introduced in [2,9]. These techniques provide advanced processing methods for determining road surface states based on radar signal information.

Along with radar signals, some research exploited video and audio signals to detect road surface conditions. A video recognition model based on the use of a support vector machine was proposed for classifying vehicle types and determining the vehicle speeds [10]. More recently, a multiple-classifier method based on video signals from a mono-camera was suggested [11]. There exists a detection technique that attempts to capture the diffuse and specular reflections from the road surface by using light emitting diode-based car headlamps to illuminate the road surface [12]. In [13], a recurrent neural network architecture was proposed for detecting road surface conditions based on the audio of tire-surface interaction.

Another line of research that aims to identify road surface conditions by exploiting electromagnetic waves has been developed, which includes the study described in [14]. Here, the authors utilized a multilayer polar-metric model for capturing the changes in the polarization of a reflected electromagnetic wave when the road surface is becoming covered with a growing ice layer. To obtain data more directly, an approach capable of detecting road surface conditions was shown, focusing on the hardware needed for the practical implementation [3]. The proposed platform uses sensors tightly integrated with vehicle electronic control units.

Additionally, some efforts at detecting road surface conditions using optical sensors have been made [15,16,17]. A Bayesian classifier for automatically identifying such conditions was developed in [7]. The authors utilized a principal component analysis to reduce the number of dimensions of the inputs from a radar system. The reduced number of radar signals represented through hidden values are then classified into a particular state based on the conditional probabilities against this state within a training dataset. Moreover, a study reported on a framework that aims to detect the changes in road surface conditions by using acceleration sensors in smartphones by calculating the variance of the vertical component of acceleration values [18].

Although many studies have been conducted that aim to automate the identification of road states by utilizing various sensor technologies, such as radar signals, video, and audio, they have mainly focused on how such technologies are able to be adapted to the identification of road surface conditions, leaving the actual models that can deal with the problem directly in a real-world setting.

## 3. Proposed Approach

### 3.1. Problem Definition and Proposed Approach

In this section, we describe the structure of a radar signal, and define the road surface detection problem considered herein by introducing symbols to formally describe the problem considered in this research. A radar system produces multiple signal values according to the number of sensors installed. The number of channels in a radar system is determined based on the amount of space needed to be covered by the system. Usually, multiple sensors are equipped sequentially in a radar system, and each sensor is responsible for sensing its own area. Therefore, such a system is considered to produce an ordered set of signals. Moreover, to examine the changes in road surface conditions, a series of radar signals over time is required because a single set of radar signals only captures the road surface state at a specific point in time.

**Figure 2:**
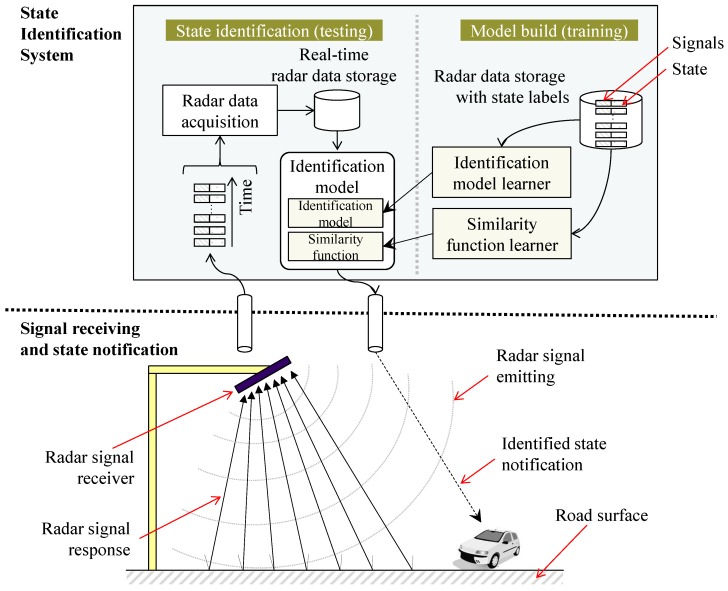
Overall framework of this research and its application scenario.

Figure 2 illustrates the overall framework of the proposed approach. The past observations, each of which consists of a pair of signal values and its corresponding state, are stored in the training database. These observations will be utilized to learn state identification functions, which are discussed in Section 3.3. The proposed framework consists of an identification model and a similarity function for collaboratively determining the current state of the road surface based on the radar signals transmitted from a radar system. The details of the identification model are discussed in Section 3.2.

**Figure 3:**
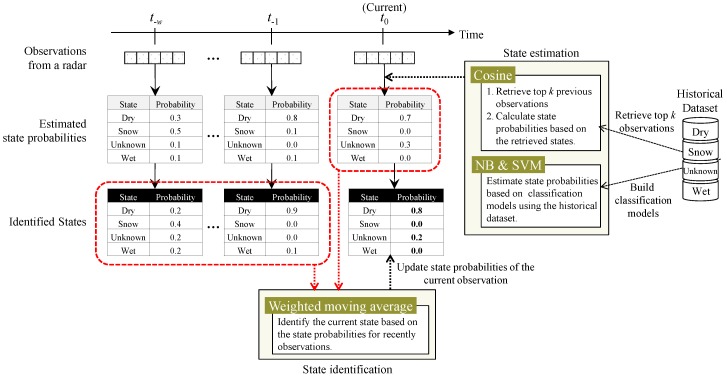
Concept of the state identification tasks in the proposed approach.

Figure 3 depicts the concept of the state identification tasks involving the state estimation and identification based on similarity functions and the weighted moving average, respectively. For the current observed signal from a radar, the probability that the observed signal is judged to be a state is calculated by utilizing the state estimation task.

The observed data from a radar system, *X*, with *n* channels during time window w, defined as:

(1) $$X=\left(\begin{array}{ccc} x_{1,1} & \cdots & x_{1,T}\\ \vdots & \ddots & \vdots \\ x_{N,1} & \cdots & x_{N,T} \end{array}\right),$$

where *N* and *T* represent the number of values observed at a particular time and the number of time slots considered in the model, respectively.

At a specific time, a set of observed data is defined in the form of an *n* dimensional vector. For instance, at time *t*, observation $x_{t}$ is denoted as ${x_{t}}^{T}=<x_{1,t},x_{2,t},\ldots ,x_{N,t}>$, and a vector is used for the input of the proposed model. Here, $x_{i,t}$ is a positive numerical value that represents the strength of a signal observed from the *i*-th radar channel at time *t*. Note that we do not consider the maximum value of a radar signal in the remainder of this paper because the detailed range of $x_{i,t}$ is dependent on the type and strength of the radar system. The obtained signal values are then normalized as $0\leq x_{i,t}\leq 1,\;i=1,\ldots ,N,\;t=1,\ldots ,T$. Additionally, we denote *S* as a set of road surface conditions, such as dry, snowy, unknown, or wet, and the actual and identified states of the road surface at time *t* are denoted as $s_{t}$ and $\hat{s_{t}}$, respectively.

### 3.2. Similarity Functions

In this section, three similarity functions for measuring how an observation is likely to be in a specific road surface state are explored. Among the previously developed similarity functions, for this research we chose cosine, naïve-Bayesian, and support vector machine-based similarity functions, respectively called COS, NB, and SVM. These similarity functions have been widely adopted in various fields of industry, such as for text documents, images, and signal information [19]. The selected similarity functions are able to produce outputs of less than a few seconds in duration during the testing phase, which is critical to the problem considered herein.

#### 3.2.1. Cosine-Based Similarity

A conventional cosine similarity function measures the proximity of two given normalized vectors based on the angle between them [20]. Therefore, it is capable of yielding satisfactory results even if the two signals have very different ranges of values. By utilizing the original cosine function, we define a similarity function of an observation with respect to a specific road surface state. Specifically, for a new observation, $x_{new}$, its cosign value against an observation, x, is defined as:

(2) $$\sigma \left(x_{new},x\right)=\frac{\sum_{i=1}^{N}x_{new,i}x_{j,i}}{\sqrt{\sum_{i=1}^{N}{\left(x_{new,i}\right)}^{2}}\sqrt{\sum_{i=1}^{N}{\left(x_{j,i}\right)}^{2}}},$$

where $x_{new}$ and x are an *N*-dimensional vector of newly-observed radar signal values and |·| represents the number of observations of a given state.

For a new observation and a state of interest, the cosine based similarity function is designed to calculate their similarity by investigating the cosine values between the new observation and each observation in the state. Then, the final similarity between a new observation and a state, $sim_{COS}\left(x_{new},s\right)$, s∈S, is obtained by averaging the top *k* similarity values among the obtained cosine values. This means that a new observation is likely to show a state as it is more similar to some of the observations in the state.

#### 3.2.2. Naïve-Bayesian-Based Similarity

We also employ the naïve-Bayesian method as a similarity function. In a naïve-Bayesian based similarity function, the similarity value of a new observation against a particular state is determined based on the conditional probability of the signal values found in the observations of this state. Bayesian-based methods have been widely adopted in various applications of sensor signal-related problems, such as quality assessments of the sensor data [21], and matching the radiometric measurements [22]. Compared to the cosine-based similarity function, it is believed that a naïve-Bayesian-based similarity function shows better results since it exploits prior knowledge based on the previous observations for each state, whereas a cosine similarity function only searches for the most similar observations for a state. In this research, we assume the observation values for each radar channel follows the normal distribution. Specifically, the similarity between a new observation, $x_{new}$, and a particular state, s∈S, is calculated as:

(3) $$sim_{NB}\left(x_{new},s\right)=\mathrm{Pr}\left(s\;|\;x_{new}\right)=\frac{\mathrm{Pr}\left(s\right)\mathrm{Pr}\left(x_{new}\;|\;s\right)}{\sum_{s^{\prime }\in S}\mathrm{Pr}\left(s^{\prime }\right)\mathrm{Pr}\left(x_{new}\;|\;s^{\prime }\right)},$$

where Pr(s), s∈S, is the probability that a randomly selected observation among all of the observations previously found in state s, and Pr(· | ·) represents the conditional probability.

In Equation (3), because a conditional probability is likely to be zero or a very small value in some cases, we regard an observation of a particular state to be the same as a new observation if the difference between their individual signal values are less than three percentage points.

#### 3.2.3. Support Vector Machine-Based Similarity

In addition to the cosine- and naïve-Bayesian-based similarity functions, a support vector machine-based function is also considered for measuring the similarity among observations [23]. A support vector machine regards an observation as a point within a multiple dimensional space, and searches for a hyper-plane that is most suitable for distinguishing the observations of each road surface state [24]. In this paper, a simple linear function, which has been widely adopted in various applications of continuous-valued classification problems, is applied as a kernel for the support vector machine.

A linear kernel-based support vector machine is defined as $\rho \left(x_{new},s_{j}\right)=w_{j}\cdot x_{new}+b_{j}$, where $w_{j}$ is the parameter in a vector form for judging state $s_{j}$, and $b_{j}$ is a slack variable for the same state. The two parameters, $w_{j}$ and $b_{j}$, can be simply obtained through estimations by the support vector machine involving the following optimization problem:

(4) $$\underset{w_{j}}{\min}\frac{∥w_{j}{∥}^{2}}{2}\;\;\mathrm{subject}\;\mathrm{to}\;\delta_{ij}\left(w_{j}\cdot x_{new}+b_{j}\right)\geq 1,\;j=1,2,\ldots ,T,$$

where $\delta_{ij},\;i=1,2,\ldots ,N,\;j=1,2,\ldots ,T$ are membership indicators such that $\delta_{ij}=1$ if the *i*-th observation is in the *j*-th state; $\delta_{ij}=0$, otherwise.

The similarity value for an observation yielded by a support vector machine similarity function is then normalized to be between zero and 1, as in the following:

(5) $$sim_{SVM}\left(x_{new},s\right)=\frac{\rho \left(x_{new},s\right)-\underset{j}{\min}\;\rho \left(x_{new},s_{j}\right)}{\underset{j}{\max}\;\rho \left(x_{new},s_{j}\right)-\underset{j}{\min}\;\rho \left(x_{new},s_{j}\right)}$$

Note that Equation (5) is for normalizing the results calculated by a support vector machine, allowing the results to be used as the probability.

#### 3.2.4. Comparison among the Similarity Functions

Table 1 compares the methodological properties of the three similarity functions considered in this research in terms of type, similarity concepts (how to measure the level of similarity for each state), and parameters. COS defines the manner of how to measure a pair of observations without any decision model. That is, it attempts to measure the similarity of a new observation against each state by simply calculating the angles among the new observation and some of the observations for each state without a learning phase. On the contrary, in the training phase, NB and SVM search for the best probability model and hyperplane, respectively, for distinguishing each state from the others in advance. Then, a new observation is investigated by using the models and hyperplanes to decide the most likely state for the new observation.

It is expected that the learning-based functions, such as NB and SVM, outperform the non-learning-based method, COS, since such learning-based functions attempt to understand the classification patterns by exploiting the entirety of training observations. For NB and SVM, NB is likely to show unsatisfactory performances compared to SVM since the independence assumption among features makes NB limited in discovering the causal relationships among features [25]. Moreover, the effective selection of the probability distribution that is unknown for the real-world settings might also prevent NB from accurately training its parameters [26].

### 3.3. State Identification

By comparing the similarity value between an observation, x, for a specific road surface state, $s_{j}\in S$, with other states, such as $s_{j^{\prime }}\in S$, where $j\neq j^{\prime }$, the most probable road surface state at a specific time can be identified. However, as previously mentioned, a similarity value yielded at a specific time needs to be calibrated by utilizing the previously identified states; otherwise, the model tends to yield non-robust outputs based on time. For instance, within only one hour, it is possible for a model to output some combination of four distinct conditions, i.e., dry, snowy, unknown, and wet, for the same area, caused by noise from receiving stable signal values.

Therefore, the proposed model finally determines the current state of a road surface at a specific time using the weighted moving average of the similarity values including the current and previous values. Specifically, the modified strength of an *i*-th radar channel at time *t* using the simple moving average, denoted as follows:

(6) $$state\left(x_{t}\right)=\underset{s\in S}{\mathrm{argmax}}\left\{\begin{array}{c} \alpha \cdot sim\left(x_{t},s\right)+\left(1-\alpha \right)\cdot sim\left(x_{t-1},s\right)\\ +{\left(1-\alpha \right)}^{2}\cdot sim\left(x_{t-2},s\right)+\ldots +{\left(1-\alpha \right)}^{w}\cdot sim\left(x_{t-w},s\right) \end{array}\right\},$$

where α is the decay factor that determines the level of influence of past observations for determining the state of the current road surface.

In Equation (6), the value of α is set to between 0.0 and 1.0. As the value of α approaches 1.0, the determination of the model is made based more on recent observations. Here, more recently observed instances have a greater effect on the current determination. On the contrary, as the value of α approaches 0.0, the determination is more dependent on past observations.

## 4. Experiments

**Figure 4:**
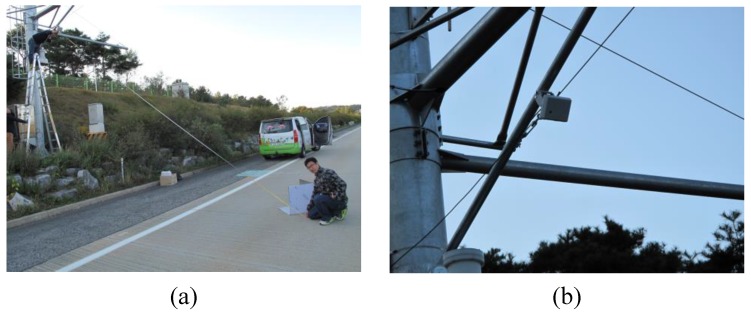
Installation of radar sensors in a highway area in South Korea.

To demonstrate the effectiveness of the proposed approach, we set up an experiment to collect radar signal information in a real-world highway area in Yeo-Su, South Korea. The highway area was dedicated to the experiments, i.e., the experiments were conducted under a minimized number of unrelated factors affecting the road surface conditions. The two images shown in Figure 4 respectively depict the installation of the radar sensors in the highway area, and an installed radar sensor that is ready to receive radar signals from the road surface and transfer them to the proposed methods.

**Figure 5:**
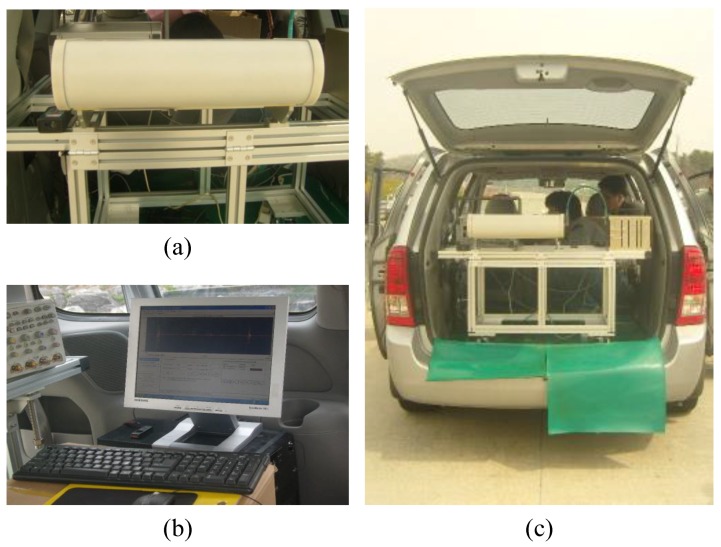
(**a**) Radar sensor hardware; (**b**) server machine equipped with the proposed models; and (**c**) radar sensor and server machine installed in a car.

Moreover, Figure 5 shows the radar hardware and software designed to be installed in a car for mobility to test the performance in various highway areas. Figure 5a shows the radar sensor hardware designed for observing the conditions of a specific road area using the refection of radio frequencies, whereas Figure 5b shows the server machine used to judge the road surface conditions by applying the proposed approach. Figure 5c shows the entire experimental setting involving both radar hardware and software.

Using the setting described above, we conducted various experiments by assuming the existence of unexpected road surfaces by applying the road surface conditions that we wanted, including dry, wet, and snowy. During a six-week period, from the first week of January 2016, to the second week of February 2016, we installed a set of radar signal equipment consisting of radar hardware and software that realized our approach. Although the radar hardware is able to collect one observation per second, at most, we collected one observation per minute because observations conducted too frequently can easy produce noise rather than improve the performance. Moreover, it is quite natural for a road surface condition in a specific area to barely change within only a few minutes.

**Figure 6:**
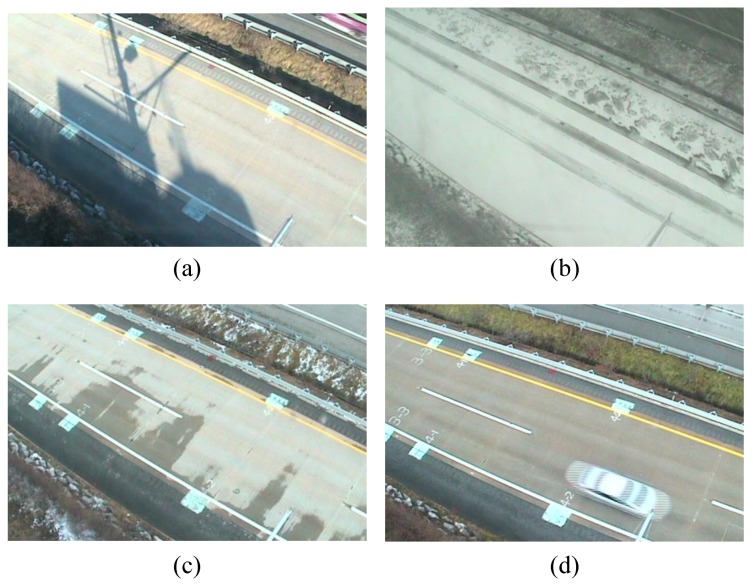
Examples of the four road surface states: (**a**) dry; (**b**) snowy; (**c**) unknown; and (**d**) wet.

A total of 115,192 observations were collected under the four states considered, i.e., dry, snowy, unknown, and wet, and the distribution of the observations are shown in Table 2. Note that an unknown state is defined when the road surface condition was recognized to have more than one state by human experts. For instance, the road surface condition was set to unknown when the snow on the road surface was starting to melt until the road surface became completely dry. Figure 6 shows photographs of the four road surface conditions considered. In detail, Figure 6a–d show a road surface under dry, snowy, unknown, and wet states, respectively.

To validate the performances of the applied methods, we also used observations for the four types, dry, snow, unknown, and wet, obtained from the test road considered in this experiments. That is, our experiments were designed to figure out how much the proposed methods precisely judge the current states of road surface conditions by utilizing the past observations. Specifically, during the last day of the data collecting period, the test observations were obtained during 482 min, 62 min, 286 min, and 50 min for dry, unknown, snow, and wet states, respectively. For the measurement criterion, we employ accuracy, which is the ratio of the correct detections among all of the detections.

Table 3 summarizes the detailed performances of the proposed methods using three similarity functions according to various values of window length, w; the number of past similar observations, k; and the amount of influence of the past observations, α. The best results of each similarity function are shown in bold. Overall, it can be concluded that SVM is the most appropriate similarity function for measuring the similarity between a newly-observed instance and a specific road surface state. In all experimental settings considered in this paper, SVM outperforms the others in terms of accuracy regardless of the applied parameter values. In addition, NB is the second-best similarity function, and it outperforms COS for most cases.

For each similarity function, the best and worst performances were demonstrated under various parameter settings. First, when COS is applied, the overall accuracy is 0.784, and the highest performances are found when α=0.7 and k=3 under w=200 or w=300, whereas the lowest performance is found when α=0.5 and k=1 under w=100. Second, when NB is used as a similarity function, the overall performance was 0.842. The highest performances are shown for the three parameter configurations, such as (1) α=0.7, k=3, and w=100; (2) α=0.9, k=3, and w=200; and (3) α=0.9, k=3, and w=300. Similar to COS, the lowest performance is achieved for α=0.5 and k=1 under w=100. Finally, when SVM is utilized as a similarity function, the overall performance is 0.898. The highest performances are observed when (1) α=0.7, k=5, and w=100 and (2) α=0.9, k=5, and w=200. Note that there are no significant changes when w and k become larger than 300 and 5, respectively.

**Figure 7:**
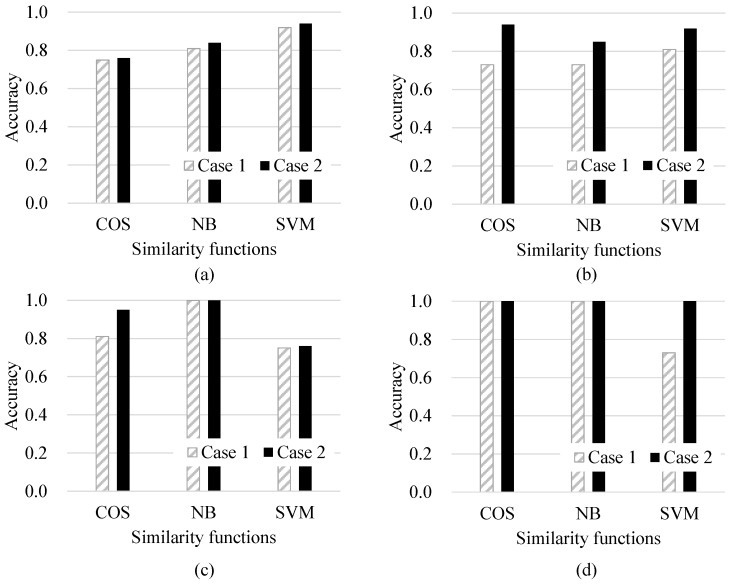
Experimental results for (**a**) dry; (**b**) snowy; (**c**) unknown; and (**d**) wet conditions under k=5 and α=0.7, and Cases 1 and 2 represent w=1 and w=100, respectively.

Figure 7 shows the effectiveness of adopting a moving average scheme to calibrate the identification results under the four representative experimental settings of the road surface conditions. Here, Cases 1 and 2 in Figure 7 are the results obtained with and without calibration using the exponential moving averages, respectively. For Case 1, w=1 indicates that the identification of the current road surface state is based only on the present judgment and without the use of past determinations. The results show that Case 2 outperforms Case 1 regardless of the similarity functions and parameter settings utilized. This implies that the use of past observations to modify the current result is successful at improving the performances of road state identification tasks.

First, for the dry state, the difference between the performances achieved by Cases 1 and 2 is quite marginal. However, Case 2 still shows higher accuracy than Case 1. Second, for the snow state, Case 2 shows greatly improved results compared to Case 1 for all the similarity functions suggested herein. Next, for the unknown state, Case 2 is successful at improving accuracy when COS is used for similarity calculation. Finally, for the wet state, Case 1 using both COS and NB show perfect accuracy, whereas SVM fails to yield satisfactory results in Case 1. However, SVM yields quite impressive results when Case 2 is applied.

**Figure 8:**
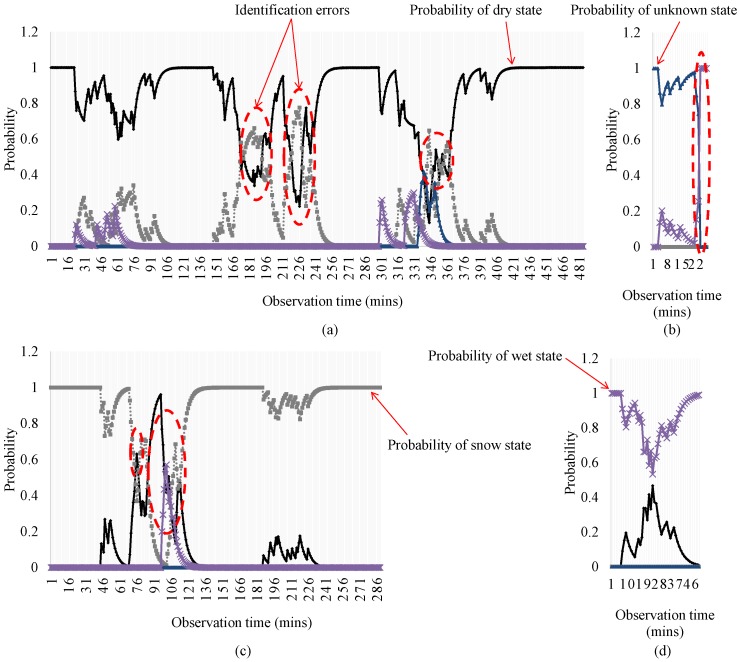
Estimated probabilities for (**a**) dry; (**b**) unknown; (**c**) snowy; and (**d**) wet conditions.

Figure 8 visualizes the changes on the estimated states over time obtained by using the proposed approach applying SVM under k=5, α=0.7, and w=100. The four graphs in Figure 8 depict how well the proposed approach determines the probabilities of the four states considered. For the dry state, shown in Figure 8a, the proposed approach generates errors at three different times, which are shown as the red dotted circles. For all of the errors, the proposed approach misjudges the current state as the snow state rather. Still, however, the probabilities of the dry state are quite high compared to those of the other states. Figure 8b,c show the estimated probabilities of each state when the actual state of a road surface are unknown and snowy, respectively. Although the proposed approach outputs robust and accurate results in most cases, it fails to yield correct identifications at three different times. For the snow state, in particular, the proposed approach misclassifies the state for quite a lengthy duration. For the wet state, as shown in Figure 8d, the proposed approach showed no error during any of the time slots considered.

Next, to quantify the robustness of the proposed approach through incorporating the weighted moving average of the similarity values for previously identified observations, we measured the robustness of a series of identifications as $1-\left(\frac{c}{t}\right)$ where c and t represent the number of changes of identification decisions and the total number of identification decisions. For instance, the robustness of a given decision for five consecutive time slots, such that $\left(s_{A},s_{A},s_{B},s_{A},s_{B}\right)$, is calculated to be $1-\left(\frac{3}{5}\right)=0.4$ since there exist three changes in the last three time slots among five decisions. By using the robustness measure, Table 4 shows the effectiveness of the usage of the weighted moving average in the proposed method. The results imply that the re-calculation of the state of an observation based on the previously identified results through utilizing the weighted moving average scheme is significantly helpful to improve both robustness and accuracy.

**Figure 9:**
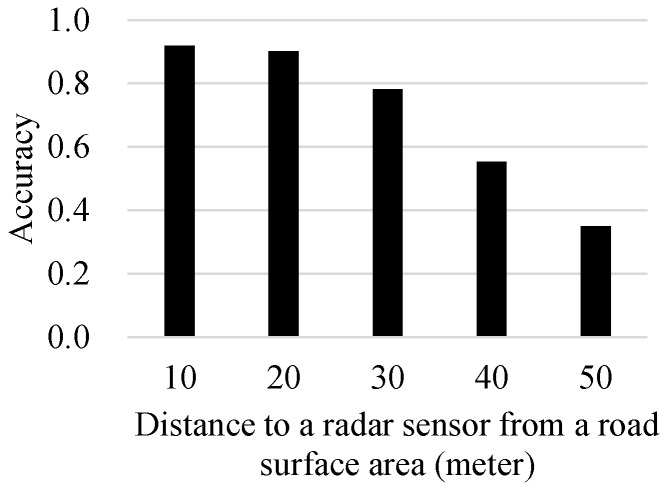
Performances according to the distance to a radar sensor from a road surface area. Note that the experiments were conducted under k=5 and α=0.7 when SVM is applied as the similarity function.

Finally, Figure 9 indicates the performance degradation of the proposed approach as the distance to a radar sensor from a road surface area of interest increases from 10 m to 50 m. The highest performances are found when the distance is 10 m, and a marginal performance degradation is observed when the distance was 20 m. However, as the distance becomes larger than 30 m, the performance rapidly decreased. It is expected that the more noise caused by sensing errors under the higher distance prevented the proposed method to yield accurate results, especially during its similarity computation task. Such inaccurately-computed similarity values may eventually accelerate wrong decisions in the re-calculation steps based on the previously-decided observations that are not reliable.

## 5. Conclusions

In this paper, an information retrieval approach for identifying road surface conditions for effective and robust performance is proposed. Specifically, machine-learning methods, such as naïve-Bayes and support vector machines, are involved to measure the similarity among newly- and previously-observed instances. Based on the similarity of results, the proposed approach judges the current state of a newly-observed instance by searching for the most related observations. The current state is then modified to be robust based on the past determinations.

The experimental results imply that the proposed approach is effective at identifying road surface conditions with respect to four states, i.e., dry, snowy, unknown, and wet. The performances achieved were better than those of conventional state identification methods that are not concerned with the application of a sophisticated similarity function or the robustness of the results in terms of time. We hope that the proposed approach will be significantly helpful at improving vehicle safety by properly observing the surface conditions of the road ahead.

Based on the findings discovered throughout this research, we plan to expand our work to hidden seasonal patterns and geographical dependencies of the results through an analysis of the historical conditions of a road surface. Moreover, we believe that the proposed method still has room for improvement. First, because sensor signals show very unstable values when objects, such as moving vehicles, are present, pre-filtering and noise reduction are required to further improve the state identification. Next, much more varying experimental conditions need to be considered. Finally, the automatically-determined conditions of a road surface will be much more valuable if a framework that can directly deliver such information to other related sub-systems can be developed.

## Figures and Tables

**Table 1:** Comparison of the methodological properties of the three similarity functions considered.

Similarity Functions	Types	Similarity Concepts	Parameters
Cosine-Based Similarity (COS)	Non-learning	The angle between a pair of vectors, new and past observations.	The number of past observations to be compared (denoted as *k*).
Naïve-Bayesian-Based Similarity (NB)	Parametric learning method	The conditional probability density of each state given new observation.	The probability parameters, mean and variation, for each radar channel.
Support Vector Machine-Based Similarity (SVM)	Non-parametric learning method	The geographical distance of new observation based on the hyperplane that separates observations according to their states.	None

**Table 2:** Distribution of road surface states in the dataset considered.

State	# Instances	% Instances
Dry	65,960	57.26%
Snowy	39,304	34.12%
Unknown	3128	2.72%
Wet	6800	5.90%
Total	115,192	100.00%

**Table 3:** Overall performance results in terms of accuracy according to the three suggested similarity functions.

w	α	COS			NB	SVM
		k=1	k=3	k=5		
100	0.5	0.762	0.781	0.782	0.842	0.895
	0.7	0.790	0.797	0.797	**0.857**	0.905
	0.9	0.773	0.789	0.789	0.844	**0.916**
200	0.5	0.767	0.777	0.782	0.843	0.894
	0.7	0.785	**0.798**	0.797	0.844	0.905
	0.9	0.773	0.778	0.789	**0.857**	**0.916**
300	0.5	0.767	0.777	0.782	0.843	0.894
	0.7	0.785	**0.798**	0.796	0.844	0.905
	0.9	0.773	0.778	0.787	**0.857**	**0.916**

**Table 4:** Robustness evaluation in terms of time under k=5 and α=0.7, and Cases 1 and 2 represent w=1 and w=100, respectively.

Measures	Without Weighted Moving Average	With Weighted Moving Average
Robustness	0.818	0.953
Accuracy	0.809	0.919
